# Prevalence of abnormal findings in 230 knees of asymptomatic adults using 3.0 T MRI

**DOI:** 10.1007/s00256-020-03394-z

**Published:** 2020-02-14

**Authors:** Laura M. Horga, Anna C. Hirschmann, Johann Henckel, Anastasia Fotiadou, Anna Di Laura, Camilla Torlasco, Andrew D’Silva, Sanjay Sharma, James C. Moon, Alister J. Hart

**Affiliations:** 1 Institute of Orthopaedics and Musculoskeletal Science, University College London and the Royal National Orthopaedic Hospital, Stanmore, Middlesex, London HA7 4LP UK; 2 Department of Radiology and Nuclear Medicine, University Hospital Basel, Basel, Switzerland; 3 Institute of Cardiovascular Science and Barts Heart Centre, University College London, London, UK; 4 Department of Cardiovascular Sciences, St George’s University of London, London, UK

**Keywords:** Knee injuries, Pain-free, Radiology, Elderly

## Abstract

**Objective:**

To identify abnormalities in asymptomatic sedentary individuals using 3.0 Tesla high-resolution MRI.

**Materials and methods:**

The cohort comprised of 230 knees of 115 uninjured sedentary adults (51 males, 64 females; median age: 44 years). All participants had bilateral knee 3.0 T MRIs. Two senior musculoskeletal radiologists graded all intraarticular knee structures using validated scoring systems. Participants completed Knee Injury and Osteoarthritis Outcome Score questionnaires at the time of the MRI scan.

**Results:**

MRI showed abnormalities in the majority (97%) of knees. Thirty percent knees had meniscal tears: horizontal (23%), complex (3%), vertical (2%), radial (2%) and bucket handle (1%). Cartilage and bone marrow abnormalities were prevalent at the patellofemoral joint (57% knees and 48% knees, respectively). Moderate and severe cartilage lesions were common, in 19% and 31% knees, respectively, while moderate and severe bone marrow oedema in 19% and 31% knees, respectively. Moderate-intensity lesion in tendons was found in 21% knees and high-grade tendonitis in 6% knees—the patellar (11% and 2%, respectively) and quadriceps (7% and 2%, respectively) tendons being most affected. Three percent partial ligamentous ruptures were found, especially of the anterior cruciate ligament (2%).

**Conclusion:**

Nearly all knees of asymptomatic adults showed abnormalities in at least one knee structure on MRI. Meniscal tears, cartilage and bone marrow lesions of the patellofemoral joint were the most common pathological findings. Bucket handle and complex meniscal tears were reported for the first time in asymptomatic knees.

**Electronic supplementary material:**

The online version of this article (10.1007/s00256-020-03394-z) contains supplementary material, which is available to authorized users.

## Introduction

Pathologies of the knee joint increase with age, and may be already existing on magnetic resonance imaging (MRI) before middle age, even without symptoms [1].

In fact, both well and poorly functioning knees can have similar damage, making it difficult to correlate relevant MRI findings with the patients’ knee pain [2–4]. Advice on permitted load and stress limits in asymptomatic knee pathologies to prevent from advancing osteoarthritis (OA) remain unclear [1].

MRI has high sensitivity for the detection of subtle changes of joint structures [5, 6]. The estimated prevalence of MRI lesions in asymptomatic knees varies significantly between studies, from 0 to 75% [2, 3]. This is due to varying study designs, including different MRI field strengths and sequences employed—indicative of variation in diagnostic accuracy [7, 8]—as well as cohorts of varying size and levels of physical activity [1].

Although 1.5 T MRI is widely clinically used, limitations have been acknowledged, particularly in evaluating abnormalities of the hyaline articular cartilage and meniscus [9–11]. Existing literature demonstrates that 3.0 T MRI provides important clinical benefits over 1.5 T, as the stronger field strength increases signal-to-noise ratio allowing improved visualisation of anatomical and pathological structures [5, 12]. Additionally, using a multichannel coil improves sensitivity and diagnostic quality [13, 14].

The purpose of this study was to determine the prevalence of abnormal knee findings in asymptomatic adults by means of a high-field strength 3.0 Tesla (T) MRI and multichannel knee coil. This is the largest study to date using this high-resolution technology to provide a robust analysis of all knee structures.

## Methods

### Study design and participants

This was a prospective cohort study including asymptomatic adults. The study received ethical approval and all volunteers provided written informed consent before participation.

We recruited 115 asymptomatic volunteers (51 males, 64 females; median age: 44 years, range 25–73 years). The study was London-based and the volunteers were 95% Welsh/English/Scottish/Northern Irish/British, of white ethnicity. Twenty-five volunteers were aged < 40 years and 90 were aged ≥ 40 years. The median body mass index (BMI) was 25 (19.6–38.1) kg/m^2^ and physical activity of low intensity was 2 (0–4) h/week. The main inclusion criteria were sedentary individuals, not meeting physical activity requirements of 30 min of moderate-intensity physical activity, 5 days/week, or 20 min of more intense physical activities, 3 days/week, based on existing health recommendations [15–17]; no present or previous history of knee injury; no prior knee surgery and asymptomatic knee joints. Pregnant women, individuals aged < 18 years, non-sedentary, with known knee problems or poor cardiovascular health were excluded from the study.

The participants were asked to complete a questionnaire called The Knee Injury and Osteoarthritis Outcome Score (KOOS) to assess their perceived knee condition and ensure that they were asymptomatic [18].

### MRI protocol

All volunteers underwent bilateral knee 3 Tesla MR (Prisma, Siemens Healthcare, Erlangen, Germany) with a dedicated 15-channel knee coil. The imaging protocol included 3 proton density–weighted fat-suppressed (PD FS) sequences in axial (repetition time/echo time [ms]: 4630/37), sagittal (4200/41) and coronal planes (5240/41). All slices were 3 mm thick, with an image size/acquisition matrix of 320 × 320 pixels. The scanning time per volunteer was 25 min in total (to scan both knees of each volunteer).

### Imaging analysis

All MR images were reviewed using a picture archiving and communications system (PACS) workstation by a senior musculoskeletal radiologist with 10-years’ experience at consultant level. Twenty percent of the cohort were randomly selected for an additional independent evaluation by a second musculoskeletal radiologist with 9-years’ experience at a consultant level.

In case of discrepancies between the radiologists’ reports concerning the findings, agreement (consensus scores) was achieved by radiologists with a consensus reading in a second MRI reporting session.

MRI findings of the knee joint were analysed using different validated scoring systems for the presence of any signal changes/lesions of varying severity for the following structures: menisci, cartilage, bone marrow, tendons, ligaments (Table 1) [3, 19–24]. Other findings were also specified, including effusion, synovial collections (prepatellar bursitis, pes anserine bursitis, Hoffa’s synovitis) and cysts (Baker’s cyst, other ganglion cysts; Table 1) [25, 26]. The scoring systems are summarised in Appendix 1 (Supplementary Materials). The patella was divided anatomically into medial and lateral regions, with the ridge being considered as part of the medial region. The tibia was divided into medial and lateral regions. The femur was divided into medial, lateral and trochlea regions and the trochlea was further divided into medial, central and lateral. The medial and lateral menisci were each divided into subregions: anterior horn and posterior horn. Scores were assigned for each individual region. All MRI abnormalities with a grade/score > 0 were counted.

**Table 1:** Grading systems for all assessed knee features on MRI

Knee feature	Grading system
Meniscus	Modified BLOKS [19] and ACLOAS [20]^†^
Cartilage	Modified Noyes and Stabler [3, 21, 22]^††^
Bone marrow	KOSS [23]
Tendons	Johnson DP et al. [24]^†††^
Ligaments	ACLOAS [20]
Joint effusion	WORMS [25]
Synovial collections*	Binary—MOAKS [26]
Iliotibial band	Binary—MOAKS [26]
Cysts**	Binary

*BLOKS*, Boston Leeds Osteoarthritis Knee Score; *ACLOAS*, Anterior Cruciate Ligament OsteoArthritis; *KOSS*, Knee Osteoarthritis Scoring System; *WORMS*, Whole-Organ Magnetic Resonance Imaging Score; *MOAKS*, MRI Osteoarthritis Knee Score. *Synovial collections: prepatellar bursitis, pes anserine bursitis, Hoffa’s synovitis; **cysts: Baker’s cyst, other ganglion cysts. ^†^Both horns of the meniscus were assessed, except for the body. ^††^A modified Noyes system on a scale 0–4 used by several papers was included here. ^†††^Scoring system primarily designed for the patellar tendon and was adjusted to include other tendons. Binary scoring system was defined as present/absent

### Statistical analysis

Comparisons between groups were performed using the unpaired *t* test, Mann–Whitney *U* test or chi-squared test respectively. Possible associations were explored by calculating odds ratios (OR) with 95% confidence intervals (CI). Statistical significance was defined as *p* < 0.05 (GraphPad Prism, version 6.0c).

## Results

Nearly all knees (227/230; [97%]) of asymptomatic individuals showed abnormalities in at least one of the knee structures on MRI, of varying grades of severity. These findings included meniscal tears, cartilage abnormalities, bone marrow oedema and tendon and ligament abnormalities. No major discrepancies between the scores of the two radiologists were reported. Mean KOOS scores for each individual item were ≥ 90/100: symptoms (90.0 ± 14.0); pain (94.9 ± 8.8); function in daily living (97.1 ± 6.5); function in sport and recreation (92.3 ± 11.6) and knee-related quality of life (90.4 ± 13.8). Further details are presented in Appendices 2 and 3 (Supplementary Materials).

### Meniscal tears: prevalence, location, type

The prevalence of asymptomatic meniscal tears was 30% in knees (Table 2). Meniscal degeneration was present in a further 18%.

**Table 2:** Prevalence of meniscal tears and degeneration in 230 asymptomatic knees

Meniscal anatomy		Number (%) of knees with meniscal abnormalities*								
		Meniscal degeneration	Meniscal extrusion	Meniscal tears						
				Horizontal	Vertical	Radial	Root	Bucket handle	Complex	Any type of tear (at least 1)
Medial	AH	2 (1%)	0 (0%)	6 (3%)	0 (0%)	1 (0.4%)	0 (0%)	0 (0%)	0 (0%)	7 (3%)
	PH	37 (16%)	5(2%)	53 (23%)	5 (2%)	5 (2%)	0 (0%)	2 (1%)	5 (2%)	70 (30%)
Lateral	AH	3 (1%)	0 (0%)	2 (1%)	0 (0%)	0 (0%)	0 (0%)	0 (0%)	1 (0.4%)	3 (1%)
	PH	5 (2%)	1 (0.4%)	2 (1%)	1 (0.4%)	0 (0%)	0 (0%)	0 (0%)	0 (0%)	3 (1%)
Any location		41 (18%)	6 (3%)	53 (23%)	5 (2%)	5 (2%)	0 (0%)	2 (1%)	6 (3%)	70 (30%)

*Grades were defined according to modified BLOKS [19] and ACLOAS [20] systems; *BLOKS*, Boston Leeds Osteoarthritis Knee Score; *ACLOAS*, Anterior Cruciate Ligament OsteoArthritis; *AH*, anterior horn; *PH*, posterior meniscal horn. The percentages do not all add up to 100% because each knee could have more than one type of meniscal abnormality and in more than one segment of the meniscus

The majority of tears were located in the medial meniscus (93%), and in its posterior horn (91%; Table 2). Lateral meniscal tears were equally found in both the posterior and anterior horns.

**Fig. 1:**
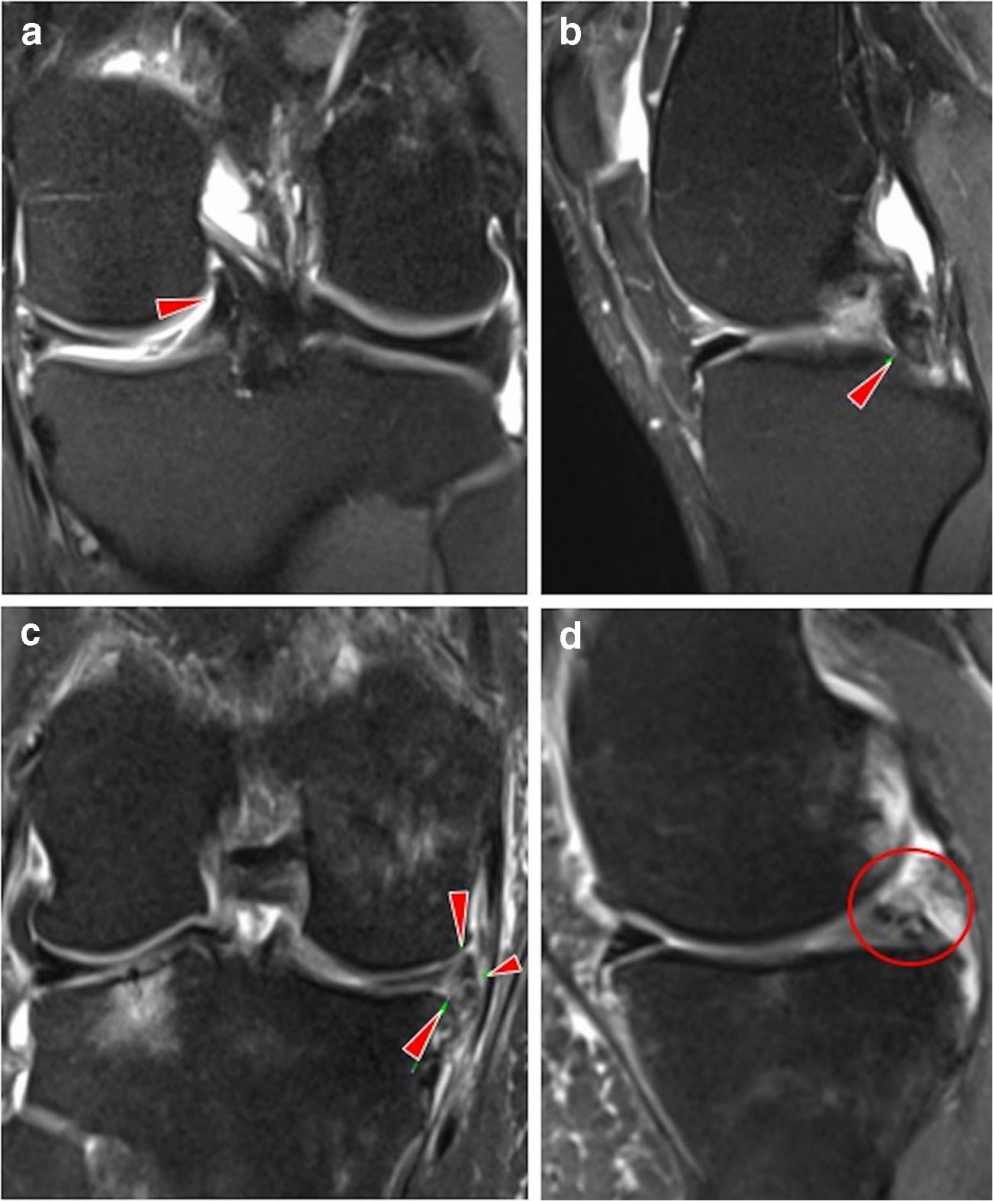
Coronal proton-density fat-saturated MR images (a, c) and sagittal images (b, d) demonstrate bucket handle tear (a, b; arrowheads) in the left knee of a 54-year-old man, and complex macerated (c, arrowheads; d, circle) meniscal tear in the right knee of a 57-year-old woman

The types of meniscal tears that we found were horizontal (23% knees), complex (3%), vertical (2%), radial (2%) and bucket handle tears (1%); meniscal extrusion was present in 3% knees (Table 2, Fig. 1).

### Articular cartilage abnormalities: prevalence, severity, location

**Fig. 2:**
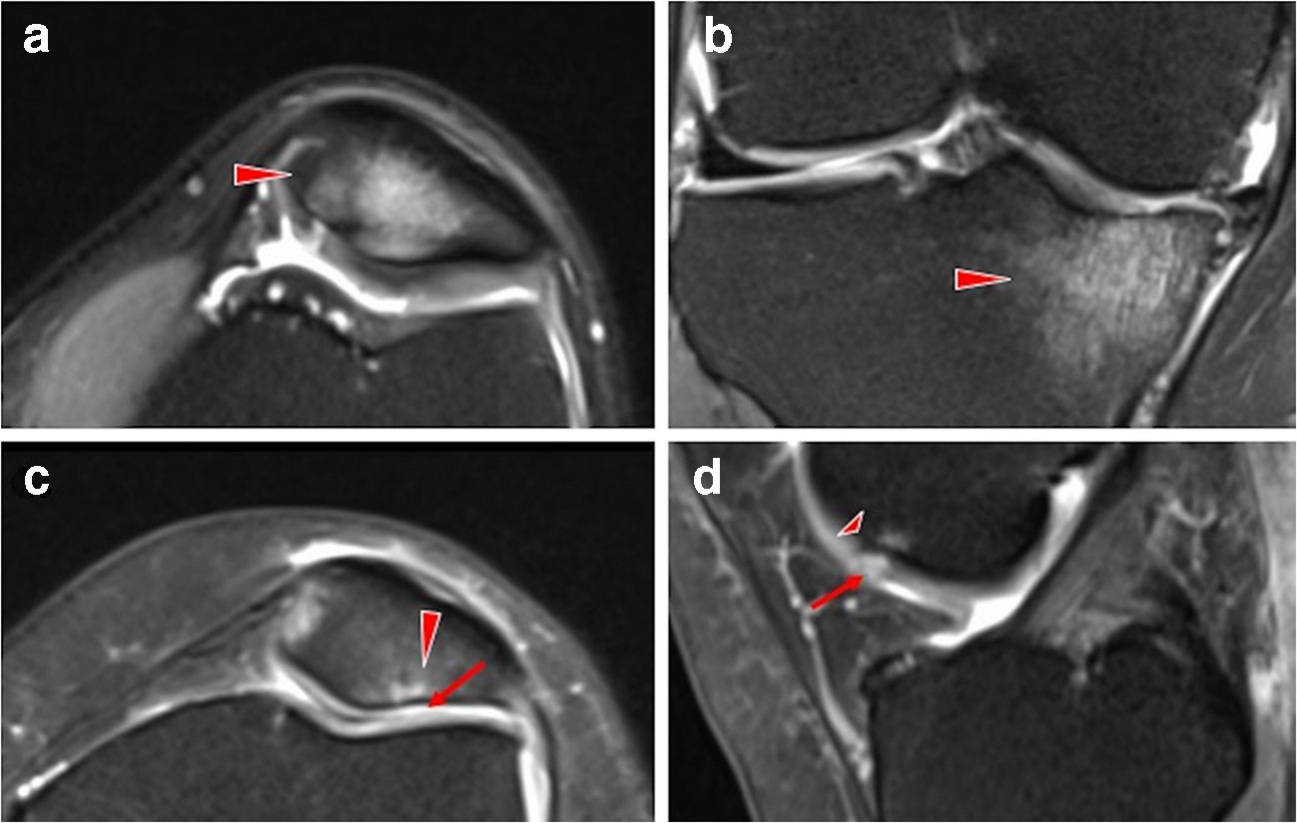
Axial proton-density fat-saturated MR images (a, c), coronal (b) and sagittal images (d) of high-grade bone marrow oedema lesion (grade 3: diameter ≥ 20 mm; in the (a) patella of the left knee of a 40-year-old man, (b) tibia of the right knee of a 59-year-old man; arrowheads) and high-grade cartilage defect (grade 4: full thickness defect exposing the bone; in the (c) patella of the left knee of a 44-year-old woman; arrow; with subchondral bone marrow oedema, arrowhead; (d) femur of the right knee of a 31-year-old woman; arrow; with subchondral ganglion cyst; small arrowhead)

Cartilage abnormalities were present in 62% of the scanned knees (Table 3). The severity of cartilage defects were as follows: 20% knees had minor grade 1 cartilage lesions, 19% knees had grade 2, 19% knees grade 3 (moderate) cartilage lesions, 31% knees grade 4 (severe) cartilage lesions (Fig. 2); 41% knees had grade 3 and/or 4 lesions (moderate/severe).

**Table 3:** Prevalence of MRI abnormalities of the articular cartilage and bone marrow in 230 asymptomatic knees

Anatomical structure	Number (%) of knees graded per structure*					
	0	1	2	3	4	Any grade ≥ 1
Cartilage						
Patellofemoral	100 (43%)	37 (16%)	32 (14%)	28 (12%)	57 (25%)	130 (57%)
Medial tibiofemoral	190 (83%)	11 (5%)	9 (4%)	6 (3%)	14 (6%)	40 (17%)
Lateral tibiofemoral	207 (90%)	9 (4%)	2 (1%)	4 (2%)	10 (4%)	23 (10%)
Any knee compartment**	87 (38%)	46 (20%)	43 (19%)	43 (19%)	71 (31%)	143 (62%)
Bone marrow						
Patellofemoral	132 (57%)	24 (10%)	39 (17%)	11 (5%)	-	98 (43%)
Medial tibiofemoral	200 (87%)	13 (6%)	14 (6%)	5 (2%)	-	30 (13%)
Lateral tibiofemoral	215 (93%)	5 (2%)	9 (4%)	2 (1%)	-	15 (7%)
Any knee compartment**	111 (48%)	42 (18%)	57 (25%)	16 (7%)	-	119 (52%)

*Grades were defined according to a modified Noyes system [3, 21, 22] for cartilage lesions and *KOSS*, Knee Osteoarthritis Scoring System [23], for bone marrow oedema; **any abnormalities in any of the knee joints. The percentages do not add up to 100% because each knee could have more than one type/grade of lesion, in more than one location. All knees with any type of lesion 1–4 were counted separately to avoid counting the same knees more than once

The patellofemoral compartment was the most affected region (57% knees).

### Bone marrow oedema: prevalence, severity, location

Bone marrow oedema–like lesions were found in 52% of the scanned knees (Table 3). By looking at levels of severity, 18% knees had only minor grade 1 bone marrow oedema lesions, 25% knees had grade 2 (moderate) oedema lesions, 7% knees had grade 3 (severe) lesions (Fig. 2) and 27% knees had grade 2 and/or 3 lesions (moderate/severe).

The region presenting with the majority of MRI changes was the patellofemoral compartment (43% knees).

### Tendon abnormalities: prevalence, severity, location

**Fig. 3:**
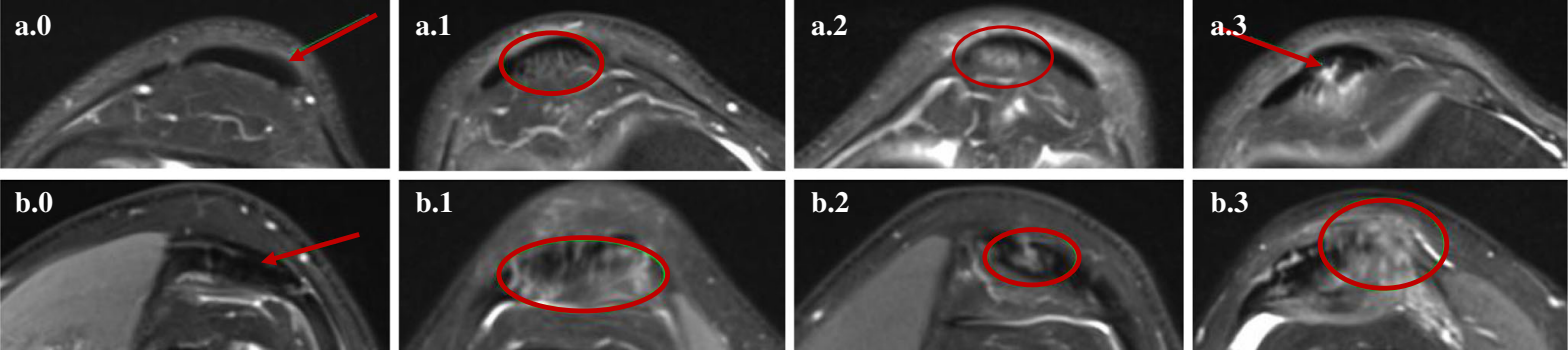
Axial proton-density fat-saturated MR images of (a) patellar tendons (a.0, grade 0; in the left knee of a 40-year-old man; a.1, grade 1; in the right knee of a 62-year-old man; a.2, grade 2; in the left knee of a 56-year-old man; a.3, grade 3; in the right knee of a 44-year-old man) and (b) quadriceps tendons (b.0, grade 0; left knee of a 40-year-old man; b.1, grade 1; in the right knee of a 40-year-old woman; b.2, grade 2; in the left knee of a 44-year-old man; b.3, grade 3; in the right knee of a 48-year-old man). The tendons are indicated by red arrows or circles; grade 0: normal tendon appearances; grade 1: increased signal intensity in less than 25% of the axial cross-sectional tendon width; grade 2: increased high-signal intensity in 25 to 50% of the axial cross-sectional tendon width; grade 3: increased high-signal intensity occupying more than 50% of the axial cross-sectional tendon width

We identified 46% knees with tendon abnormalities (Table 4). In terms of levels of severity, 22% knees had only minor increased signal intensity (grade 1), 21% knees had grade 2 moderate signal intensity lesions and 6% knees had grade 3 lesions/high-grade tendonitis (Fig. 3). MRI signal changes were most visible in the patellar tendon (27% knees), followed by the quadriceps tendon (13% knees).

**Table 4:** Prevalence of MRI abnormalities of the knee tendons and ligaments of 230 asymptomatic knees

Anatomical structure	Number (%) of knees graded per structure*				
	0	1	2	3	Any grade ≥ 1
Tendons					
Patellar	169 (73%)	30 (13%)	26 (11%)	5 (2%)	61 (27%)
Quadriceps	201 (87%)	9 (4%)	16 (7%)	4 (2%)	29 (13%)
Semimembranosus	207 (90%)	11 (5%)	9 (4%)	3 (1%)	23 (10%)
Sartorius	228 (99%)	1 (0.4%)	0 (0%)	1 (0.4%)	2 (1%)
Gracilis	222 (97%)	4 (2%)	0 (0%)	4 (2%)	8 (3%)
Any tendon	124 (54%)	51 (22%)	48 (21%)	14 (6%)	106 (46%)
Ligaments					
Anterior cruciate	151 (66%)	75 (33%)	4 (2%)	0 (0%)	79 (34%)
Posterior cruciate	228 (99%)	1 (0.4%)	1 (0.4%)	0 (0%)	2 (1%)
Medial collateral	224 (97%)	4 (2%)	2 (1%)	0 (0%)	6 (3%)
Lateral collateral	227 (99%)	3 (1%)	0 (0%)	0 (0%)	3 (1%)
Any ligament	143 (62%)	81 (35%)	7 (3%)	0 (0%)	87 (38%)

*Grades were defined according to Johnson DP et al. [24] for tendon abnormalities and *ACLOAS*, Anterior Cruciate Ligament Osteoarthritis Score [20], for ligamentous abnormalities. The percentages do not add up to 100% because each knee could have more than one type/grade of lesion, in more than one location. All knees with any type of lesion 1–3 were counted separately to avoid counting the same knees more than once

### Ligamentous abnormalities: prevalence, severity, location

We found 38% knees (Table 4) with ligamentous abnormalities. In terms of levels of severity, 35% knees had only a thickened ligament (grade 1) and 3% knees had grade 2/partial rupture. No grade 3 injuries were identified.

The anterior cruciate ligament was the most affected ligament among the participants (34% knees), with the other ligaments presenting only very few lesions (Table 4).

### Prevalence of other findings

Joint effusion was found in 3% knees: grade 2 (*n* = 7) and grade 3 (*n* = 1).

Other findings included Baker’s cyst (33% knees), prepatellar bursitis (26% knees), Hoffa’s synovitis (23% knees), other ganglion cysts (20% knees) and pes anserine bursitis (6% knees).

### Associations between lesions

There was an association between the presence of abnormal cartilage signal and bone marrow oedema in knees (*p* < 0.0001). Participants with cartilage abnormalities were 8.0 times more likely to have bone marrow oedema lesion (95% CI, 1.6–10.3; *p* = 0.0023). No associations were found for other lesions (*p* > 0.005; Appendix 4 (Supplementary Materials)).

### Participant characteristics

No difference in the prevalence of MRI abnormalities between males and females was found.

The prevalence of lesions generally increased with age. The mean age for the participants with a meniscal tear was slightly higher than those without a tear (47.5 ± 9.9 years (*n* = 50) vs 42.6 ± 7.0 (*n* = 65); *p* = *p* = 0.0027, unpaired *t* test). The mean age for those with bone marrow oedema was slightly higher than those without oedema (46.4 ± 8.9 years (*n* = 72) vs 42.0 ± 7.8 (*n* = 43); *p* = *p* = 0.0071, unpaired *t* test). Participants aged ≥ 40 years old were 4.0 times more likely to have abnormal cartilage signal (95% CI, 1.6–10.3; *p* = 0.0023). In terms of level of severity, 51 of 90 participants (57%) aged ≥ 40 years had high grade 3 or 4 cartilage lesions. And 10 of 25 participants (40%) aged < 40 had grade 3 or 4 cartilage lesion. The difference was not statistically significant (*p* = 0.140, chi-squared). The distribution of prevalences per knees is available in Table 5.

**Table 5:** Number of participants with both knees or single knees showing abnormalities on MRI, respectively, and total number of knees affected, in those aged < 40 and ≥ 40, respectively, in the meniscus, articular cartilage, bone marrow, tendons and ligaments

Key knee abnormalities	Participants (%) with both knees affected	Participants (%) with single knees affected		Total knees (%) affected		
		Right knee	Left knee	Right knee	Left knee	All knees
Aged < 40 (*N* = 25, 50 knees)						
Meniscal tears	2 (8%)	4 (16%)	0 (0%)	6 (12%)	2 (4%)	8 (16%)
Cartilage abnormalities	7 (28%)	3 (12%)	2 (8%)	10 (20%)	9 (18%)	19 (38%)
Bone marrow oedema	8 (32%)	3 (12%)	2 (8%)	11 (22%)	10 (20%)	21 (42%)
Tendon abnormalities	5 (20%)	6 (24%)	2 (8%)	11 (22%)	7 (14%)	18 (36%)
Ligament abnormalities	3 (12%)	7 (28%)	1 (4%)	10 (20%)	4 (8%)	14 (28%)
Aged ≥ 40 (*N* = 90, 180 knees)						
Meniscal tears	21 (23%)	4 (4%)	16 (18%)	25 (14%)	37 (20%)	62 (34%)
Cartilage abnormalities	54 (60%)	10 (11%)	6 (7%)	64 (36%)	60 (33%)	124 (69%)
Bone marrow oedema	39 (43%)	12 (13%)	8 (9%)	51 (28%)	47 (26%)	98 (54%)
Tendon abnormalities	26 (29%)	20 (22%)	16 (18%)	46 (26%)	42 (23%)	88 (49%)
Ligament abnormalities	25 (28%)	9 (10%)	14 (16%)	34 (19%)	39 (22%)	73 (41%)

The BMI of participants with MRI abnormalities was not significantly different from those without abnormalities, except for tendon abnormalities (*p* = 0.0002). The odds of a participant with BMI ≥ 25 kg/m^2^ (overweight) presenting with a tendon abnormality were 3.3 (95% CI, 1.5–7.6). A total of 28 of 60 participants (47%) with BMI ≥ 25 kg/m^2^ had grade 2 or 3 high-intensity tendonitis (Fig. 3); 18 of 55 participants (33%) with BMI < 25 kg/m^2^ showed high-grade tendon lesion (the difference was not statistically significant, *p* = 0.128, chi-squared).

## Discussion

Overall our study showed a high prevalence of 3.0 T MRI pathologies in the knees of asymptomatic adults: meniscal tears, including few complex and bucket handle tears; patellofemoral cartilage lesions and bone marrow oedema lesions of moderate to severe grade. The prevalences were higher than in previous studies. The KOOS results confirmed that the participants had no perceived knee problems/symptoms of functional limitation, despite the observed lesions on MRI.

### Previous studies in asymptomatic uninjured knees

A number of studies have reported prevalences of knee abnormalities in uninjured asymptomatic individuals. Culvenor et al. [1] collated in a recent systematic review the pooled results from the existing evidence.

The first interesting finding is the prevalence of meniscal tears. While 44 studies (3761 knees from 2817 participants) reported prevalence of meniscal tears with an overall pooled prevalence estimate of 10% (95% CI 7 to 13%; *I*^2^ = 87.2%) [1], we hereby reported a significantly higher prevalence of 30%. Moreover, we identified vertical, radial, bucket handle and complex tears which are not common in asymptomatic individuals [27]. Therefore, they may be clinically more meaningful.

In terms of cartilage defects (partial and full thickness), 42 studies (4322 knees from 3446 participants) reported an overall pooled prevalence estimate of 24% (95% CI 15 to 34%; *I*^2^ = 97.8%) [1]. Our study however showed a higher prevalence that exceeds this interval: 41% cartilage defects of moderate to severe damage, with grade 4 lesions being most prevalent in asymptomatic adults (31% knees). The clinical significance of this is uncertain, raising questions about the factors leading to cartilage damage and what mechanisms of pathology prevention could be employed.

Thirty-four studies (4089 knees from 3255 participants) reported bone marrow lesions prevalence with an overall pooled prevalence estimate of 18% (95% CI 12 to 24%) [1]. In comparison with this data, our study showed a slightly higher prevalence of 27% moderate to severe bone marrow oedema–like lesions. Clinically, this may be of importance as bone marrow lesions are linked to the onset of osteoarthritis [28–30].

Prevalence of ligament tears was 0% for 16 of the 20 studies, with the remaining four studies reporting 1–30% of mostly anterior cruciate or collateral ligament partial tears [1]. Similarly, our results showed no complete tears and a low prevalence of 3% partial ligamentous tears, of the anterior cruciate and lateral collateral ligaments.

Regarding asymptomatic knee tendon abnormalities, there is not much evidence in the literature about their incidence. Matiotti SB et al. [31] identified 19.5% tendon injuries in asymptomatic soccer players—adolescents—and we identified a prevalence of 26% cases of tendon abnormalities in our study. The observation of asymptomatic patellar tendonitis may suggest that this type of injury could result in future symptoms future and encourages closer monitoring of these cases [31–34].

The prevalence of lesions was reported to increase with age [1]; this is in agreement with our study outcomes. Also we showed that overweight people are more predisposed to load-bearing tendon thickness, finding which is supported by previous studies [35–39].

### Study strengths and limitations

The main study strengths are the large sample size, the methodology employed in the study (3.0 T MRI and multichannel coil) and the detailed analysis of knee structures. As compared with the clinically widely used 1.5 T system, 3.0 T MRI reported higher diagnostic confidence for better visualisation of the morphology and pathology of joint structures [5, 6, 40]. Also, the multichannel technology offers additional benefits of higher spatial resolution and increased diagnostic quality [13, 14]. So far 11 studies have employed the 3.0 T MRI technique for the assessment of knee structures and the sample size did not exceed 95 asymptomatic knees in any MRI trial [41–51]. This study involves the highest number of knees that were ever scanned with 3.0 T MRI, in particular of asymptomatic sedentary older adults. Additionally, we did an in-depth analysis of all structures and reported the prevalence of lesions by levels of severity instead of reporting only the abnormalities irrespective of grade.

We acknowledge the following limitations: (1) MRI double-reporting was done for 20% of the cohort; however, no major discrepancies between the radiologists’ reports were identified in this subset of images so the single-reporting of the remaining scans was considered to be reliable; (2) the KOOS questionnaires, the history of any past joint problems and the activity levels of volunteers were self-reported; therefore, a risk of bias needs to be considered; (3) the analysis was confined to one ethnic group, thus limiting the potential generalisation of the findings; (4) meniscal assessment included both meniscal horns, except for the body; therefore, few lesions could have been missed; (5) follow-up studies are needed to investigate the clinical relevance of the findings over time.

## Conclusions and clinical significance

Our study questions clinical decision-making regarding arthroscopy and its efficacy in reducing symptoms and treatment. The high rate of asymptomatic adults with knee joint abnormalities on MRI may indicate why arthroscopy and other surgical interventions for these do not result in better outcomes than sham surgery [1, 52]. For example, there is no evidence to suggest that meniscectomy benefits patients presenting with meniscal tear symptoms more than sham surgery does [53]. Moreover, meniscectomy and other surgical interventions could lead to further complications or deterioration of the articular cartilage and increase the risk of osteoarthritis [54–56].

Despite the increasing use of high-resolution MRI, in practice, diagnosis should be primarily based on patient’s medical history and physical examination by an experienced clinician, instead of solely focusing on the MRI results. The images may assist in correlating clinical signs and symptoms but should not replace clinical evaluation [57, 58].

Our MRI findings can represent early signs of osteoarthritis and the clinical implications need to be investigated further, including follow-up studies over time, to inform efforts to diagnose and treat knee problems across the lifespan. Further studies could monitor whether the knee condition of those participants with lesions will progress at a faster rate over time than that of those without abnormalities. The findings may guide closer surveillance and prevent future injuries.

## Electronic supplementary material

ESM 1 (DOCX 31 kb)

ESM 2 (DOCX 21 kb)

ESM 3 (DOCX 29 kb)

ESM 4 (DOCX 391 kb)
