# Supplementary material for: Prevalence of abnormal findings in 230 knees of asymptomatic adults using 3.0 T MRI
Source: Skeletal Radiol. 2020 Feb 14;49(7):1099–107. doi: 10.1007/s00256-020-03394-z (PMC7237395; doi:10.1007/s00256-020-03394-z)
Supplement: Supplementary file 1 — (DOCX 31 kb) [file 256_2020_3394_MOESM1_ESM.docx]

| **Scoring system per knee structure** | **Scores** | |
| --- | --- | --- |
| **Modified BLOKS 0-7 [1] & ACLOAS 0-8 [2]:**  **Meniscus (medial, lateral)**  2 areas: AH, PH. | BLOKS  **Meniscal signal (not a tear)**  0=Absent  1=Present  **Type of tear:**  2=Vertical tear  3=Horizontal & radial tear  4=Complex tear  5=Root tear  6=Complete maceration  7=Meniscal cyst | ACLOAS  0=Normal meniscus with absence of tear, maceration and hypointense signal  1=Intrameniscal hyperintensity not extending to meniscal surface  2=Horizontal tear  3=Radial and vertical tear  4=Bucket-handle tear, displaced tear (including root tears) and complex tears  5=Meniscal repair  6=Partial meniscectomy and partial maceration  7=Progressive partial maceration or re-partial meniscectomy (i.e., loss of morphological substance of the meniscus) as compared to the previous visit  8=Complete maceration or resection |
| **Modified Noyes 0-4** [3–5]:  **Cartilage**  MFC, LFC, MTC, LTC, trochlea (medial, lateral, central), patella (medial, lateral) | 0=Normal 1=Grade I lesion: have areas of heterogenous signal intensity on fat saturated IW FSE sequences 2=Grade II lesion: cartilage defects that involve <1/2 of cartilage thickness 3=Grade III lesion: cartilage defects that involve >1/2 of cartilage thickness but < full thickness  4=Grade IV lesion: full thickness cartilage defects exposing the bone | |
| **KOSS 0-3** [6]**:**  **Bone marrow**  MFC, LFC, MTC, LTC, trochlea (medial, lateral, central), patella (medial, lateral) | **Bone marrow lesion** 0=absent 1=minimal (d<5mm) 2=moderate (d=5-20mm) 3=severe (d≥20mm) | |
| **Johnson DP 0-3** [7]:  **Tendinopathy (patellar, quadriceps, sartorius, gracilis)** | 0=Grade 0: Normal tendon appearances 1=Grade 1: Increased signal intensity in less than 25% of the axial cross-sectional tendon width 2=Grade 2: Increased high-signal intensity in 25% to 50% of the axial cross-sectional tendon width 3=Grade 3: Increased high-signal intensity occupying more than 50% of the axial cross-sectional tendon width | |
| **ACLOAS 0-3** [2]:  **Ligaments**  ACL, PCL, MCL, LCL | **ACL & PCL**  0=Normal ligament with hypointense signal and regular thickness and continuity 1=Thickened ligament and/or high intraligamentous signal with normal course and continuity 2=Thinned or elongated but continuous ligament  3=Absent ligament or complete discontinuity | **MCL & LCL**  0=Continuous ligament with normal signal, no surrounding hyperintensity/oedema  1=Continuous ligament with normal signal, surrounding hyperintensity reflecting edema and/or hematoma  2=Partial rupture/discontinuity with some preserved fibers  3=Complete disruption |
| **MOAKS 0-1** [8]:  **ITB signal** | 0=absent  1=present | |
| **WORMS 0-3** [9]**:**  **Joint effusion** | 0=absent  1=<33% of maximum potential distention 2=33%–66% of maximum potential distention 3=>66% of maximum potential distention | |
| **MOAKS 0-1** [8]:  **Prepatellar bursitis**  **Pes anserine bursitis**  **Baker's/popliteal cyst**  **Other ganglion cysts** | 0=absent  1=present | |
| **MOAKS 0-3** [8]**:**  **Hoffa’s synovitis** | 0=absent 1=mild 2=moderate 3=severe | |
| **WORMS 0-1** [9]:  **PMC & PLC injury** | 0=absent  1=present | |

BLOKS, Boston Leeds Osteoarthritis Score; ACLOAS, Anterior Cruciate Ligament OsteoArthritis Score; AH, anterior horn of the meniscus; PH, posterior horn of the meniscus; MFC, medial femoral condyle; LFC, lateral femoral condyle; MTC, medial tibial condyle; LTC, lateral tibial condyle; KOSS, Knee Osteoarthritis Scoring System; ACL, anterior cruciate ligament; PCL, posterior cruciate ligament; MCL, medial collateral ligament; LCL, lateral collateral ligament; MOAKS, MRI Osteoarthritis Knee Score; ITB, iliotibial band; WORMS, Whole-Organ Magnetic Resonance Imaging score; PMC, posteromedial corner; PLC, posterolateral corner.

**References**

1. Hunter DJ, Lo GH, Gale D, Grainger AJ, Guermazi A, Conaghan PG (2008) The reliability of a new scoring system for knee osteoarthritis MRI and the validity of bone marrow lesion assessment: BLOKS (Boston-Leeds Osteoarthritis Knee Score). Ann Rheum Dis 67:206–211

2. Roemer FW, Frobell R, Lohmander LS, Niu J, Guermazi A (2014) Anterior cruciate ligament osteoarthritis score (ACLOAS): Longitudinal MRI-based whole joint assessment of anterior cruciate ligament injury. Osteoarthr Cartil 22:668–682

3. Noyes FR, Stabler CL (1989) A system for grading articular cartilage lesions at arthroscopy. Am J Sports Med 17:505–513

4. Pappas GP, Vogelsong MA, Staroswiecki E, Gold GE, Safran MR (2016) Magnetic Resonance Imaging of Asymptomatic Knees in Collegiate Basketball Players: The Effect of One Season of Play. Clin J Sport Med. doi: 10.1097/JSM.0000000000000283

5. Gold GE, Chen CA, Koo S, Hargreaves BA, Bangerter NK (2009) Recent advances in MRI of articular cartilage. Am J Roentgenol. doi: 10.2214/AJR.09.3042

6. Kornaat PR, Ceulemans RYT, Kroon HM, Riyazi N, Kloppenburg M, Carter WO, Woodworth TG, Bloem JL (2005) MRI assessment of knee osteoarthritis: Knee Osteoarthritis Scoring System (KOSS) - Inter-observer and intra-observer reproducibility of a compartment-based scoring system. Skeletal Radiol 34:95–102

7. Johnson DP, Wakeley CJ, Watt I (1996) Magnetic resonance imaging of patellar tendonitis. J Bone Jt Surg - Ser B. doi: 10.1302/0301-620x.78b3.0780452

8. Hunter DJ, Guermazi A, Lo GH, Grainger AJ, Conaghan PG, Boudreau RM, Roemer FW (2011) Evolution of semi-quantitative whole joint assessment of knee OA: MOAKS (MRI Osteoarthritis Knee Score). Osteoarthr Cartil 19:990–1002

9. Peterfy CG, Guermazi A, Zaim S, et al (2004) Whole-organ magnetic resonance imaging score (WORMS) of the knee in osteoarthritis. Osteoarthr Cartil 12:177–190
