# Supplementary material for: Prevalence of abnormal findings in 230 knees of asymptomatic adults using 3.0 T MRI
Source: Skeletal Radiol. 2020 Feb 14;49(7):1099–107. doi: 10.1007/s00256-020-03394-z (PMC7237395; doi:10.1007/s00256-020-03394-z)
Supplement: Supplementary file 2 — (DOCX 21 kb) [file 256_2020_3394_MOESM2_ESM.docx]

**Appendix 2. Prevalence of MRI abnormalities of the cartilage and bone marrow in 230 knees per structure (knee compartment subdivisions)**

| **Anatomical structure** | | **Number (%) of knees graded per structure*** | | | | | |
| --- | --- | --- | --- | --- | --- | --- | --- |
|  |  | **0** | **1** | **2** | **3** | **4** | **Total 1-4** |
| **Cartilage** | |  | | | | |  |
| Patella | Medial | 144 (63%) | 19 (8%) | 16 (7%) | 17 (7%) | 34 (15%) | 86 (37%) |
|  | Lateral | 159 (69%) | 15 (7%) | 15 (7%) | 11 (5%) | 30 (12%) | 71 (31%) |
| Trochlea | Medial | 225 (98%) | 0 (0%) | 0 (0%) | 3 (1%) | 2 (1%) | 5 (2%) |
|  | Central | 218 (95%) | 0 (0%) | 2 (1%) | 3 (1%) | 7 (3%) | 12 (5%) |
|  | Lateral | 224 (97%) | 0 (0%) | 1 (0.4%) | 0 (0%) | 5 (2%) | 6 (3%) |
| Femur | Medial | 193 (84%) | 11 (5%) | 8 (3%) | 6 (3%) | 12 (5%) | 37 (16%) |
|  | Lateral | 211 (92%) | 6 (2%) | 1 (0.4%) | 4 (2%) | 8 (3%) | 19 (8%) |
| Tibia | Medial | 222 (97%) | 1 (0.4%) | 1 (0.4%) | 0 (0%) | 6 (3%) | 8 (3%) |
|  | Lateral | 221 (96%) | 4 (2%) | 1 (0.4%) | 1 (0.4%) | 3 (1%) | 9 (4%) |
| **Bone marrow** | |  | | | | |  |
| Patella | Medial | 192 (83%) | 13 (6%) | 19 (8%) | 6 (3%) | - | 38 (17%) |
|  | Lateral | 190 (83%) | 12 (5%) | 20 (9%) | 8 (3%) | - | 40 (17%) |
| Trochlea | Medial | 226 (98%) | 0 (0%) | 4 (2%) | 0 (0%) | - | 4 (2%) |
|  | Central | 225 (98%) | 0 (0%) | 4 (2%) | 1 (0.4%) | - | 5 (2%) |
|  | Lateral | 222 (97%) | 3 (1%) | 4 (2%) | 1 (0.4%) | - | 8 (3%) |
| Femur | Medial | 207 (90%) | 10 (4%) | 11 (5%) | 2 (1%) | - | 23 (10%) |
|  | Lateral | 222 (97%) | 2 (1%) | 6 (3%) | 0 (0%) | - | 8 (3%) |
| Tibia | Medial | 213 (93%) | 5 (2%) | 8 (3%) | 4 (2%) | - | 17 (7%) |
|  | Lateral | 219 (95%) | 4 (2%) | 5 (2%) | 2 (1%) | - | 11 (5%) |

*Grades were defined according to a modified Noyes system [1–3] for cartilage lesions and KOSS, Knee Osteoarthritis Scoring System [4], for bone marrow oedema.

**References**

1. Noyes FR, Stabler CL (1989) A system for grading articular cartilage lesions at arthroscopy. Am J Sports Med 17:505–513

2. Pappas GP, Vogelsong MA, Staroswiecki E, Gold GE, Safran MR (2016) Magnetic Resonance Imaging of Asymptomatic Knees in Collegiate Basketball Players: The Effect of One Season of Play. Clin J Sport Med. doi: 10.1097/JSM.0000000000000283

3. Gold GE, Chen CA, Koo S, Hargreaves BA, Bangerter NK (2009) Recent advances in MRI of articular cartilage. Am J Roentgenol. doi: 10.2214/AJR.09.3042

4. Kornaat PR, Ceulemans RYT, Kroon HM, Riyazi N, Kloppenburg M, Carter WO, Woodworth TG, Bloem JL (2005) MRI assessment of knee osteoarthritis: Knee Osteoarthritis Scoring System (KOSS) - Inter-observer and intra-observer reproducibility of a compartment-based scoring system. Skeletal Radiol 34:95–102
