# Supplementary material for: Prevalence of abnormal findings in 230 knees of asymptomatic adults using 3.0 T MRI
Source: Skeletal Radiol. 2020 Feb 14;49(7):1099–107. doi: 10.1007/s00256-020-03394-z (PMC7237395; doi:10.1007/s00256-020-03394-z)
Supplement: Supplementary file 3 — (DOCX 29 kb) [file 256_2020_3394_MOESM3_ESM.docx]

| **Anatomical structure** | **Number (%) of participants graded per knee structure*** | | | | | |
| --- | --- | --- | --- | --- | --- | --- |
|  | **0** | **1** | **2** | **3** | **4** | **Total 1-4** |
| **Cartilage** |  | | | | | |
| Patellofemoral | 35  (30%) | 25  (22%) | 28  (24%) | 25  (22%) | 36  (31%) | 80  (70%) |
| Medial tibiofemoral | 87  (76%) | 9  (8%) | 9  (8%) | 6  (5%) | 10  (9%) | 28  (24%) |
| Lateral tibiofemoral | 97  (84%) | 9  (8%) | 2  (2%) | 4  (3%) | 10  (9%) | 18  (16%) |
| Any knee compartment** | 33  (29%) | 37  (32%) | 37  (32%) | 37  (32%) | 45  (39%) | 82  (71%) |
| **Bone marrow** |  | | | | | |
| Patellofemoral | 54  (47%) | 23  (20%) | 30  (26%) | 8  (7%) | - | 61  (53%) |
| Medial tibiofemoral | 92  (80%) | 12  (10%) | 12  (10%) | 4  (3%) | - | 23  (20%) |
| Lateral tibiofemoral | 102  (89%) | 5  (4%) | 7  (6%) | 2  (2%) | - | 13  (11%) |
| Any knee compartment** | 63  (55%) | 38  (33%) | 42  (37%) | 16  (14%) | - | 52  (45%) |

**Appendix 3a. Prevalence of MRI abnormalities of the articular cartilage and bone marrow in 115 asymptomatic participants**

*Grades were defined according to a modified Noyes system [1–3] for cartilage lesions and KOSS, Knee Osteoarthritis Scoring System [4], for bone marrow oedema;**Any abnormalities in any of the knee joints. ^The percentages do not add up to 100% because each participant could have more than one type/grade of lesion in their knees, in more than one location. All participants with any type of lesion 1-4 were counted separately to avoid counting the same participant more than once.

**Appendix 3b. Prevalence of MRI abnormalities of the tendons and ligaments in 115 asymptomatic participants**

| **Anatomical structure** | **Number (%) of participants graded per knee structure*** | | | | |
| --- | --- | --- | --- | --- | --- |
|  | **0** | **1** | **2** | **3** | **Total 1-3** |
| **Tendons** |  | | | | |
| Patellar | 68  (59%) | 27  (23%) | 21  (18%) | 4  (3%) | 47  (41%) |
| Quadriceps | 90  (78%) | 9  (8%) | 15  (13%) | 2  (2%) | 25  (22%) |
| Semimembranosus | 96  (83%) | 11  (10%) | 9  (8%) | 3  (3%) | 19  (17%) |
| Sartorius | 113  (98%) | 1  (1%) | 0  (0%) | 1  (1%) | 2  (2%) |
| Gracilis | 109  (95%) | 4  (3%) | 0  (0%) | 3  (3%) | 6  (5%) |
| Any tendon | 41  (36%) | 46  (40%) | 40  (35%) | 8  (7%) | 74  (64%) |
| **Ligaments** |  | | | | |
| Anterior cruciate | 61  (53%) | 52  (45%) | 4  (3%) | 0  (0%) | 54  (47%) |
| Posterior cruciate | 113  (98%) | 1  (1%) | 1  (1%) | 0  (0%) | 2  (2%) |
| Medial collateral | 110  (96%) | 4  (3%) | 2  (2%) | 0  (0%) | 5  (4%) |
| Lateral collateral | 112  (97%) | 3  (3%) | 0  (0%) | 0  (0%) | 3  (3%) |
| Any ligament | 57  (50%) | 54  (47%) | 7  (6%) | 0  (0%) | 58  (50%) |

*Grades were defined according to Johnson DP et al [5] for tendon abnormalities and ACLOAS, Anterior Cruciate Ligament Osteoarthritis Score [6], for ligamentous abnormalities. The percentages do not add up to 100% because each participant could have more than one type/grade of lesion in their knees, in more than one location. All participants with any type of lesion 1-3 were counted separately to avoid counting the same participant more than once.

**References**

1. Noyes FR, Stabler CL (1989) A system for grading articular cartilage lesions at arthroscopy. Am J Sports Med 17:505–513

2. Pappas GP, Vogelsong MA, Staroswiecki E, Gold GE, Safran MR (2016) Magnetic Resonance Imaging of Asymptomatic Knees in Collegiate Basketball Players: The Effect of One Season of Play. Clin J Sport Med. doi: 10.1097/JSM.0000000000000283

3. Gold GE, Chen CA, Koo S, Hargreaves BA, Bangerter NK (2009) Recent advances in MRI of articular cartilage. Am J Roentgenol. doi: 10.2214/AJR.09.3042

4. Kornaat PR, Ceulemans RYT, Kroon HM, Riyazi N, Kloppenburg M, Carter WO, Woodworth TG, Bloem JL (2005) MRI assessment of knee osteoarthritis: Knee Osteoarthritis Scoring System (KOSS) - Inter-observer and intra-observer reproducibility of a compartment-based scoring system. Skeletal Radiol 34:95–102

5. Johnson DP, Wakeley CJ, Watt I (1996) Magnetic resonance imaging of patellar tendonitis. J Bone Jt Surg - Ser B. doi: 10.1302/0301-620x.78b3.0780452

6. Roemer FW, Frobell R, Lohmander LS, Niu J, Guermazi A (2014) Anterior cruciate ligament osteoarthritis score (ACLOAS): Longitudinal MRI-based whole joint assessment of anterior cruciate ligament injury. Osteoarthr Cartil 22:668–682
