# Supplementary material for: Prevalence of abnormal findings in 230 knees of asymptomatic adults using 3.0 T MRI
Source: Skeletal Radiol. 2020 Feb 14;49(7):1099–107. doi: 10.1007/s00256-020-03394-z (PMC7237395; doi:10.1007/s00256-020-03394-z)
Supplement: Supplementary file 4 — (DOCX 391 kb) [file 256_2020_3394_MOESM4_ESM.docx]

**Appendix 4. Associations between key outcomes: Number of knees with concomitant different types of abnormalities (Venn diagrams a, b; Table A4)**


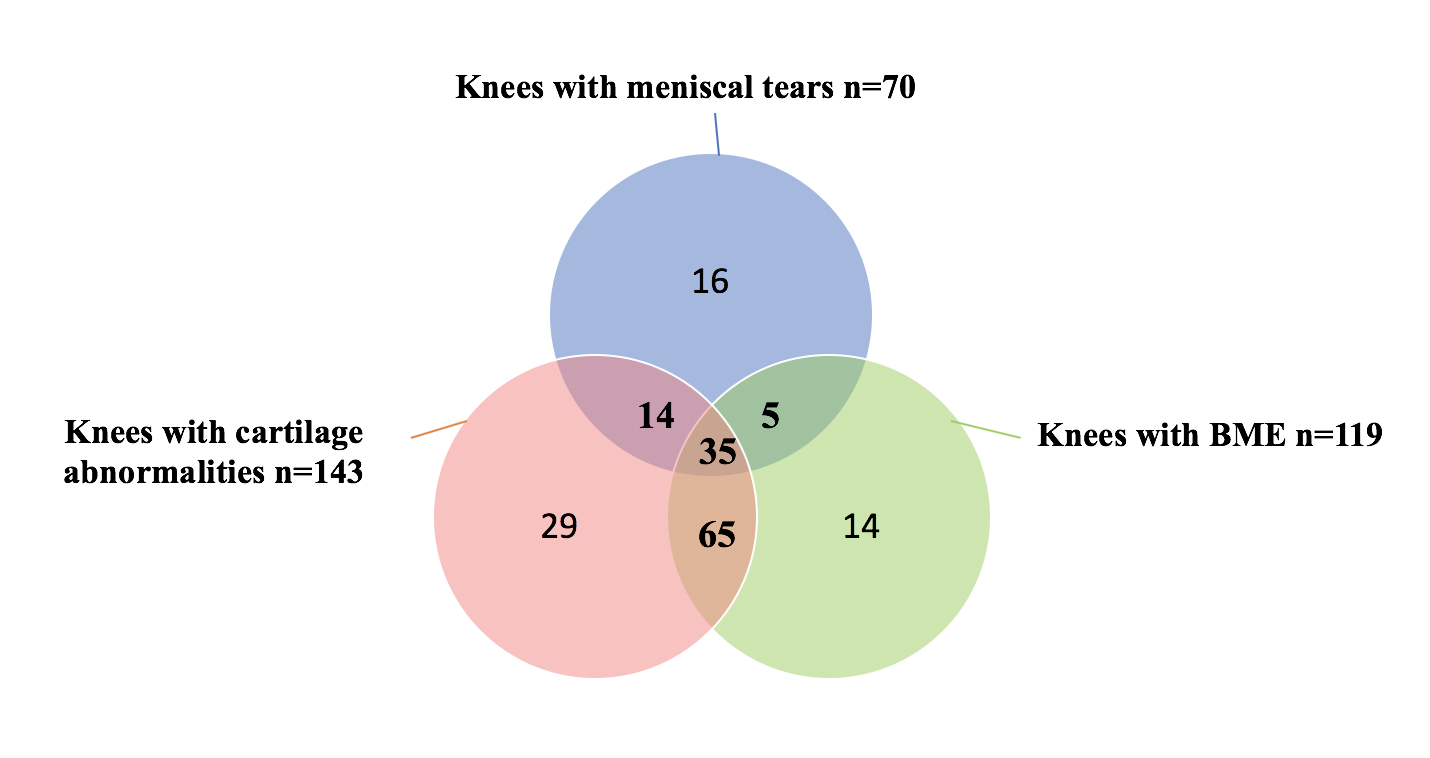


b.

a.


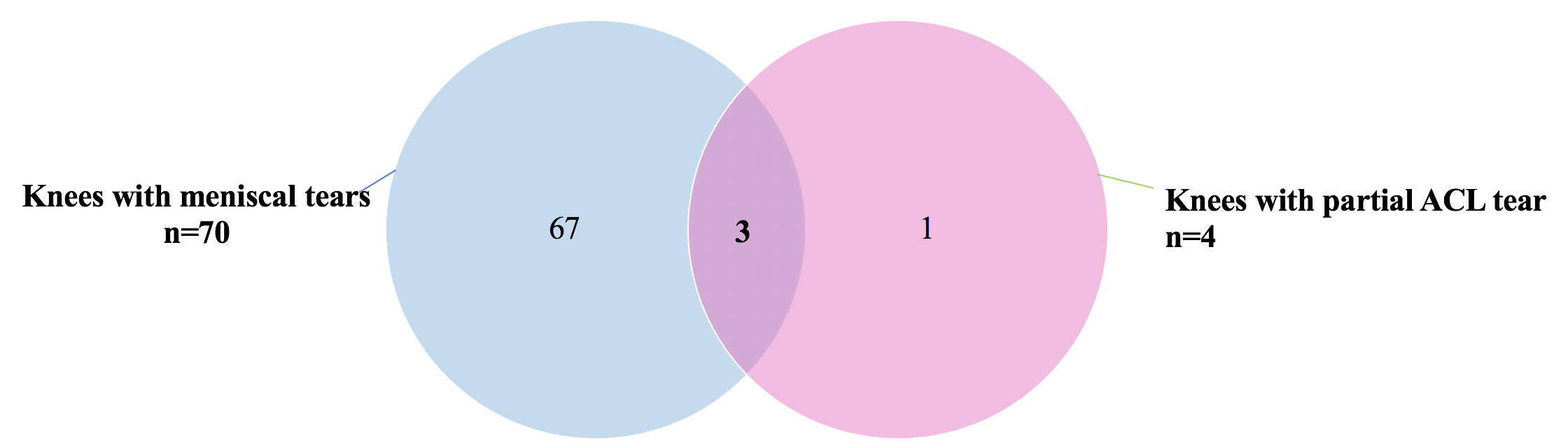


**Table A4: Concomitant abnormalities in 230 knees**

| **Types of concomitant abnormalities** | **Number of concomitant abnormalities** |
| --- | --- |
| Knees with meniscal tears and cartilage abnormalities (no BME) | 14 |
| Knees with meniscal tears and BME (no cartilage abnormalities) | 5 |
| Knees with cartilage abnormalities and BME (no meniscal tears) | 65 |
| Knees with meniscal tears, cartilage abnormalities and BME | 35 |
| Knees with meniscal tears and ACL rupture | 3 |

ACL, anterior cruciate ligament; BME, bone marrow oedema.
